# Structure and Properties of Heterometallics Based on Lanthanides and Transition Metals with Methoxy-β-Diketonates

**DOI:** 10.3390/molecules27238400

**Published:** 2022-12-01

**Authors:** Vladislav V. Krisyuk, Samara Urkasym Kyzy, Tatyana V. Rybalova, Ilya V. Korolkov, Mariya A. Grebenkina, Alexander N. Lavrov

**Affiliations:** 1 Nikolaev Institute of Inorganic Chemistry, SB RAS, Lavrentiev Ave. 3, 630090 Novosibirsk, Russia; 2 Vorozhtsov Novosibirsk Institute of Organic Chemistry, SB RAS, Lavrentiev Ave. 9, 630090 Novosibirsk, Russia; 3 Novosibirsk National Research State University, 1 Pirogova Str., 630090 Novosibirsk, Russia

**Keywords:** heterometallic complexes, coordination polymer, lanthanides, beta-diketonates, volatility, ligand exchange, molecular magnetism

## Abstract

The possibility of obtaining volatile polynuclear heterometallic complexes containing lanthanides and transition metals bound by methoxy-β-diketonates was studied. New compounds were prepared by cocrystallization of monometallic complexes from organic solvents. Ln(tmhd)_3_ were used as initial monometallic complexes (Ln = La, Pr, Sm, Gd, Tb, Dy, Lu; tmhd = 2,2,6,6-tetramethylheptane-3,5-dionate) in combination with TML_2_ in various ratios (TM = Cu, Co, Ni, Mn; L: L^1^ = 1,1,1-trifluoro-5,5-dimethoxypentane-2,4-dionate, L^2^ = 1,1,1-trifluoro-5,5-dimethoxy-hexane-2,4-dionate, L^3^ = 1,1,1-trifluoro-5-methoxy-5-methylhexane-2,4-dionate). Heterometallic complexes of the composition [(LnL_2_tmhd)_2_TM(tmhd)_2_] were isolated for light lanthanides Ln= La, Pr, Sm, Gd, and L= L^1^ or L^2^. By single crystal XRD, it has been established that heterometallic compounds containing La, Pr, Cu, Co, and Ni are isostructural linear coordination polymers of alternating mononuclear transition metal complexes and binuclear heteroleptic lanthanide complexes, connected by donor–acceptor interactions between oxygen atoms of the methoxy groups and transition metal atoms. A comparison of powder XRD patterns has shown that all heterometallic complexes obtained are isostructural. Havier lanthanides Ln = Tb, Dy, Lu did not form heterometallics. Instead, homometallic complexes Ln(L^3^)_3_ were identified for Ln = Dy, Lu as well as for Ln = La. The thermal properties of the complexes were investigated by TG-DTA and vacuum sublimation tests. The heterometallic complexes were found to be not volatile and decomposed under heating to produce inorganic composites of TM oxides and Ln fluorides. In contrast, Ln(L^3^)_3_ is volatile and may be sublimed in a vacuum. Results of magnetic measurements are discussed for several heterometallic and homometallic complexes.

## 1. Introduction

Ln-containing heterometallic complexes have attracted the attention of researchers due to their interesting magnetic, optical, and luminescent properties [1,2,3,4,5]. A significant number of works are devoted to the study of the magnetic properties of **4**f–**3**d complexes. There are also works where **4**f–**3**d heterometallic complexes exhibit several unusual properties simultaneously [1]. In addition, **4**f–**3**d complexes are studied as precursors for mixed oxides of f-d metals [6,7,8]. The latter motivates the search for new volatile polynuclear complexes with organic ligands.

**Figure 1:**
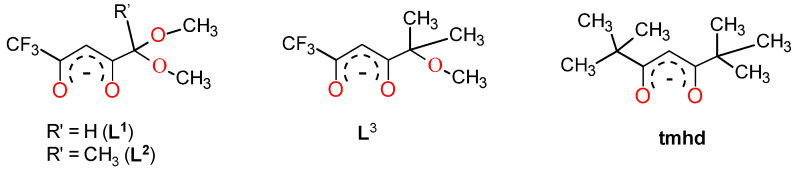
Graphical representation of the used β-diketonate ligands: L^1^ = 1,1,1-trifluoro-5,5-di-methoxypentane-2,4-dionate; L^2^ = 1,1,1-trifluoro-5,5-dimethoxyhexane-2,4-dionate; L^3^ = 1,1,1-tri-fluoro-5-methoxy-5-methylhexane-2,4-dionate; tmhd = 2,2,6,6-tetramethylheptane-3,5-dionate.

In particular, heterometallic β-diketonates usually consist of monometallic moieties sharing several chelating oxygen atoms between coordination centers and fluorinated ligands provide more volatile compounds [9]. The bridging function of oxygen atoms is limited by the steric action of the terminal substituents (R, R’) of the β-diketonate ligand R-CO-CH-CO-R’. To overcome this problem, one can use ligands with donor atoms at the terminal substituents that are more sterically accessible to form bridging bonds with neighboring metal atoms, which should be coordination unsaturated. Our systematic screening of mono- and di-methoxy-substituted diketones, including the ones presented in Figure 1 (R-CO-CH-CO-C(OCH_3_)_x_(CH_3_)_3−x_, x = 1, 2), allowed to prepare various heterometallic complexes by cocrystallization of monometallic complexes from organic solvents [10,11,12]. In the structure of such heterometallic complexes, the monometallic fragments are held together by bridging oxygen atoms, including those from methoxy groups. For example in Cu/Pb and Pd/Pb heterocomplexes, it was shown that the structure and reactivity depend on the composition and structure of ligands in the initial monometallic complexes [10,11]. First-row transition metals with methoxy-substituted diketones form a series of coordination saturated binuclear complexes that are rather stable in solution and, therefore, are inert to the formation of heterometallic products [12]. In the structure of copper (II) complexes with dimethoxy ligands and heterocomplexes based on them, usually, one of the adjacent methoxy groups does not form bridging bonds and remains free, creating only a steric effect. In order to involve two methoxy groups in one terminal substituent for bridging bonds, it is necessary to investigate the possibility of obtaining volatile heterometallic complexes with the participation of metal ions, which stand out for large coordination numbers, e.g., alkaline earth metals and lanthanides.

In this paper, we investigate the possibility of obtaining heterometallic complexes of lanthanides and first-row transition metals with mono- and dimethoxy-substituted diketonate ligands (Figure 1). We discuss here the preparation and purification methods, structure, thermal, and magnetic properties of new compounds using the example of typical representatives of a number of available lanthanides (III), namely La, Pr, Sm, Gd, Tb, Dy, Lu.

So far, for dimethoxy-substituted lanthanide diketonates, the structure of homometallic ([La(L^2^)_3_]_2_) and heterometallic complexes with alkali metals has been reported, where the coordination of metal cations is also stabilized due to bridging bonds involving methoxy groups, about half of which remain free [13,14,15,16]. The isolated compounds exhibit discrete and polymeric structures, but volatility was not reported.

## 2. Results and Discussion

### 2.1. Synthesis and Structure

**Scheme 1:**
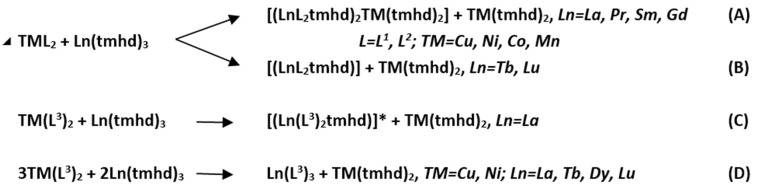
Main reactions of Ln(tmhd)_3_ and TML_2_ in organic solvent.

**Figure 2:**
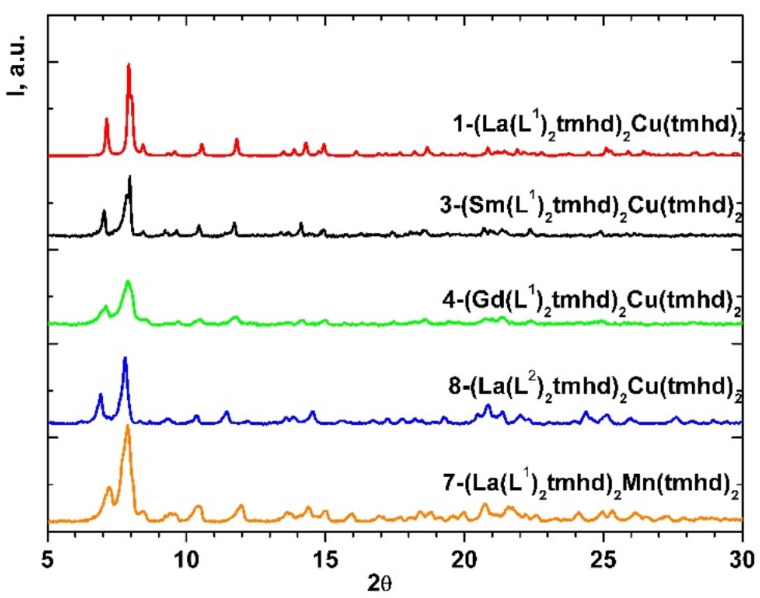
Comparison of experimental powder X-ray diffraction patterns of several heterometallic diketonates.

Previously, it was shown that the monometallic complexes Cu(L^1^)_2_, Cu(L^2^)_2_, and Cu(L^3^)_2_ have the structure of coordination polymers and heterometallic complexes based on them can have both discrete and polymeric structures where the monometallic units are held together by bridging metal–oxygen bonds with the participation of methoxy groups [10]. For dimethoxy-substituted diketonate (L = L^1^, L^2^), one of the adjacent methoxy groups in the terminal substituent remains free. To involve both methoxy groups in the formation of bridging bonds, the lanthanide complexes were used in which the central atoms can have large coordination numbers. We studied the crystalline reaction products of available Ln(tmhd)_3_ (Ln = La, Pr, Sm, Gd, Tb, Dy, Lu) with transition metal bis-diketonates, TML_2_, L = L^1^, L^2^, L^3^ (Figure 1), TM = Cu, Ni, Co, Mn. It was found that the ligand exchange reactions according to Scheme 1 occur in solution:

Heterometallic complexes were obtained only by reaction (**A**) in the series of light lanthanides and dimethoxy-substituted diketonates at a molar ratio of 1:1. Other molar ratios did not give new products. The cocrystallization product consists of two types of crystals easily distinguishable by color. Heterocomplexes of the [(LnL_2_tmhd)_2_TM(tmhd)_2_] type are slightly colored depending on the TM. Crystals of the second component TM(tmhd)_2_ could be separated either by extraction with heptane or by vacuum sublimation. For example, [(La(L^1^)_2_tmhd)_2_Cu(tmhd)_2_] is nonvolatile and non-soluble in heptane, while dark blue crystals of Cu(tmhd)_2_ are well soluble in heptane and sublime at T_subl_ = 130 °C in dynamic vacuum ~10^−2^ Torr. TM(tmhd)_2_ can easily be identified by XRD, MP, and color. The remaining heterometallic product can be recrystallized from toluene. A series of copper–lanthanide complexes [(Ln(L^1^)_2_tmhd)_2_Cu(tmhd)_2_], where Ln = La (**1**), Pr (**2**), Sm (**3**), Gd (**4**), are apparently isostructural according to powder XRD data (Figure 2). To trace the sterical effect of the terminal bis-methoxy substituent, [(La(L^2^)_2_tmhd)_2_Cu(tmhd)_2_] (**8**) was prepared. According to XRD, **8** is isostructural with the rest of the copper-containing heterocomplexes **1**–**4**. Following the same approach, [(La(L^1^)_2_tmhd)_2_Ni(tmhd)_2_] (**6**), [(La(L^1^)_2_tmhd)_2_Co(tmhd)_2_] (**5**) were isolated. Crystals suitable for single crystal XRD were not obtained for **3** and **4** and for [(La(L^1^)_2_tmhd)_2_Mn(tmhd)_2_] (**7**) complexes, the manganese derivative turned out to be very unstable during recrystallization.

**Figure 3:**
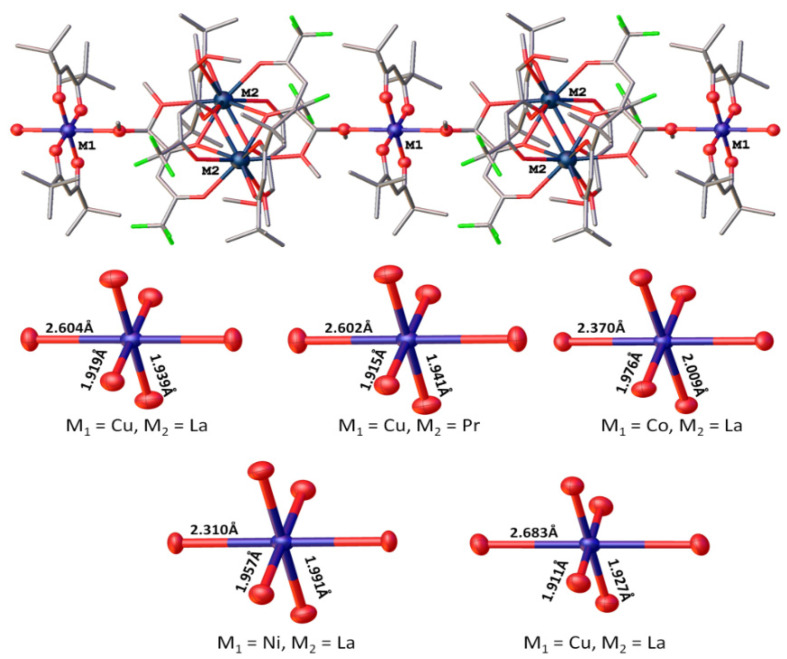
A fragment of the chain in the structure of **1**, **2**, **5**–**6**, and **8** (hydrogen atoms are omitted for clarity) with a detailed view of the TM coordination environment, respectively.

Crystals of **1**, **2**, **5**, **6**, and **8** are built of one-dimensional coordination polymers of alternating mononuclear transition metal complexes and binuclear heteroleptic lanthanide complexes, connected by donor–acceptor interactions between oxygen atoms of methoxy groups and transition metal atoms (Figure 3). In other words, the metal–organic chains are based on lanthanide binuclear fragments and transition metal fragments bridged by TM-O^O-La linkers when one of the adjacent methoxy-group is connected to Ln and another one is connected to TM atoms. As a result, the square planar coordination in TM(tmhd)_2_ fragment is completed to square-bipyramidal by two oxygen atoms of OCH_3_-groups. Key geometry parameters are summarized in Table 1.

In the binuclear heteroleptic fragment (Ln(L)_2_tmhd)_2_ for lanthanide atoms C.N. = 10. Distance Ln…Ln is ~3.8 Å. The lanthanide dimer itself is centrosymmetric and is bound by bridging bonds with the participation of oxygen atoms from both chelate and methoxy groups. However, for the two adjacent methoxy groups, only one is coordinated by the lanthanide atom. While the other methoxy-group remains free in one L, and, in the second L, is coordinated by a transition metal atom. Where both methoxy groups are coordinated the Ln-OCH_3_ distance is shorter for L^1^ in contrast to L^2^. At the same time, the TM-OCH_3_ bridging bond in **8** is longer than in **1**, apparently due to the fact that the –CCH_3_(OCH_3_)_2_ substituent in L^2^ is bulkier than –CH(OCH_3_)_2_ in L^1^. In turn, the chains of the coordination polymer do not form additional contact with each other. For example, in crystals of **2**, the shortest distance between the nearest chains is F…H = 2.620 Å (Figure S1).

In route (**B**) for the heavier lanthanides (Ln = Tb-Lu), no heterometallic products formed. In this case, Cu(tmhd)_2_ and a colorless glassy product, perhaps a mixed-ligand lanthanide complex, formed separately.

Thus, for ligands with two methoxy groups, the formation of two series of products is observed during cocrystallization. Light lanthanides (Ln = La-Gd) give isostructural heterometallic complexes with ligand exchange, while for heavier lanthanides (Ln = Tb-Lu) the ligand exchange occurs without the formation of a heterometallic complex.

**Figure 4:**
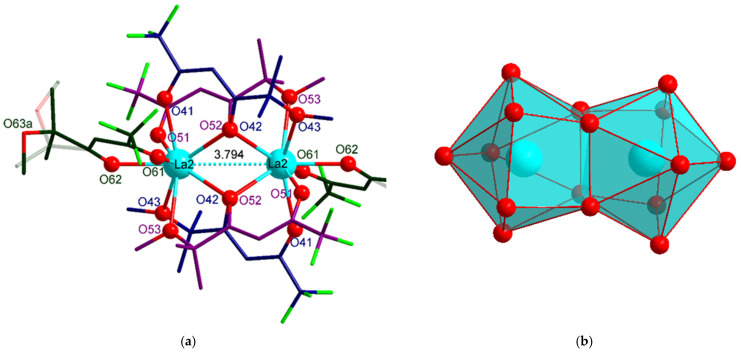
Binuclear structure of one of [La(L^3^)_3_]_2_ (**9**) molecules (**a**) and its coordination polyhedra representing the LaO_10_ coordination environment (**b**).

In the case of the mono-methoxy-substituted ligand, the situation turned out to be quite different. It was assumed that the lanthanide dimer (Ln(L^3^)_2_tmhd)_2_ should contain two methoxy groups involved in bridging bonds and none free; no heterometallic complex would form. Indeed, the verification of systems with L^3^ according to route (**C**) of Scheme 1 has shown that the cocrystallization of Cu(L^3^)_2_ and La(tmhd)_3_ taken in an equimolar ratio does not yield a heterometallic complex, but rather a two-component system of individual complexes of lanthanum and transition metal was obtained in the result of ligand exchange. The resulting mixture was separated using gradient sublimation. There was only one lanthanum-containing fraction. ^1^H NMR analysis of the lanthanum-containing fraction has shown the presence of signals for the ligands L^3^ and tmhd in a ratio of 2:1. It is difficult to say whether this is a mixed-ligand complex or a mixture of the lanthanum homoligand complexes. That is why this product is marked by an asterisk in Scheme 1. Further, it was assumed that in the result of the complete exchange of ligands, the lanthanum dimer should contain two free methoxy groups. However, the reaction (**D**) where reactants Ln(tmhd)_3_ and TML_2_ were taken in 2:3 ratio still did not give heterometallic complexes. As a result of complete exchange of ligands, homoleptic complexes of lanthanum and copper were obtained, which were also separated by vacuum sublimation. ^1^H NMR analysis of the lanthanum-containing fraction have shown the presence of signals only for the L^3^ ligands at the same place, and again two sets of signals are visible in a ratio of 1:2. Moreover, in the ^19^F NMR spectra, two signals have been recorded in a ratio of 1:2. Thus, the NMR study supports the dimeric structure of the lanthanum complex in solution, with the broad lines indicating that dimer **9** has rather a fluctional structure. Structural data also confirm the dimeric structure, with **9** containing two free methoxy groups. The structure contains two crystallography-independent molecules differing in the orientation of the terminal methoxy groups (Figure 4). The coordination of lanthanum atoms in the homoleptic dimer is similar to that for the heteroleptic dimers described above in **1**–**6**, **8**. The main bond lengths and angles are given in Table 2. There are the same coordination manner, the same coordination polyhedron, C.N. = 10, number of bridging bonds is 4. La…La distance in dimer ~3.8 Å. A similar dimeric complex [La(L^2^)_3_]_2_ has been reported before [16]. For comparison, in La_2_(tmhd)_6_, the La…La distance is ~4 Å, and there are only two bridging oxygen atoms, C.N.(La) = 7 [17].

Thus, it has been established that under the cocrystallization of La(III) dipaloylmethanate with mono-methoxy-substituted transition metal(II) β-diketonates, the formation of heterometallic complexes does not occur. Instead, an exchange of ligands is observed with the isolation of the binuclear homoleptic lanthanum complex. In the structure of **9**, the free methoxy groups are located as if along the dimer and steric conditions seem to be unfavorable for the formation of bridging bonds with square planar TM(tmhd)_2_. Nevertheless, cocrystallization in such systems can be used as a simple way to obtain complexes of some lanthanides with fluorine-containing diketones by ligand exchange reaction. We believe that for other light lanthanides Ln = La-Gd complexes have dimeric structure [Ln(L^3^)_3_]_2_. As for the heavier lanthanides, the structure of Tb, Dy, and Lu derivatives were not determined because they were isolated as glassy substances. For example, the ^1^H NMR spectrum of **11** is silent and the ^19^F NMR spectrum looks strange, indicating that this complex is paramagnetic. On the other hand, ^1^H NMR of **12** shows broad lines but for one set of ligand signals, and an asymmetric multiplet is observed in the ^19^F NMR spectrum. Thus, it can be said that at least in solution the Ln(L^3^)_3_ for Ln = Tb-Lu seem to be low-symmetry monomers. However, they are more volatile, since **11** and **12** can be re-sublimated at ~160 °C, and **9** already at ~200 °C. To determine the nuclearity of the resulting noncrystalline complexes, we additionally used data on magnetic properties, which are discussed in the corresponding section.

### 2.2. Thermal Properties

**Figure 5:**
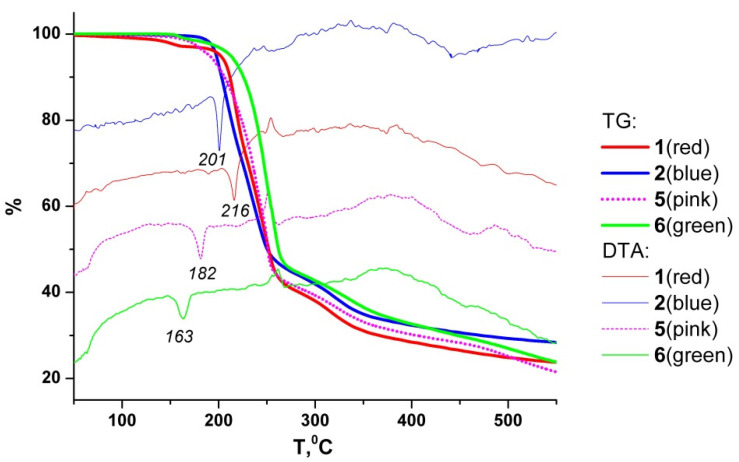
TG curves of heterometallics **1**, **2**, **5**, and **6**.

The sublimation test showed that the obtained heterometallic complexes **1**–**6**, and **8** are not volatile and decompose when heated in a vacuum. A comparison of the TG-DTA data for **1**, **2**, **5**, and **6** is shown in Figure 5. As can be seen, the heterometallic complexes exhibit similar thermal behavior, they decompose upon heating. XRD of the solid residues obtained in separate experiments on the calcination of heterocomplex samples in air showed that a composite containing CuO, LaF_3_, and LaOF is obtained from [(La(L^1^)_2_tmhd)_2_Cu(tmhd)_2_] (**1**) (Figure S2). [(La(L^1^)_2_tmhd)_2_Ni/Co(tmhd)_2_] (**5**, **6**) gives LaF_3_, LaOF and probably metallic Ni or Co. Thermolysis of [(La(L^1^)_2_tmhd)_2_Mn(tmhd)_2_] (**7**) gives solid product containing LaMnO_3_ and LaF_3_ (Figures S3–S5). As already mentioned, Ln(L^3^)_3_ complexes are volatile and can be re-sublimed in a vacuum.

**Figure 6:**
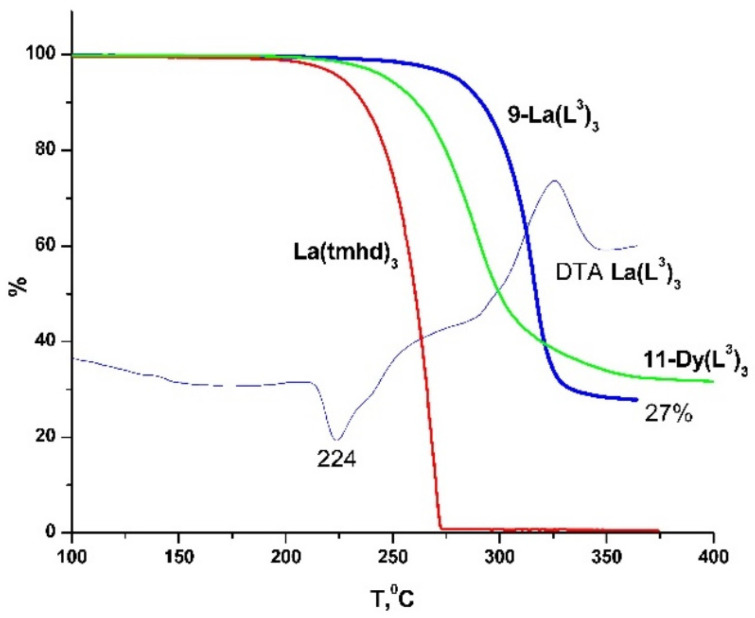
Comparison of TG data for homometallic tris-diketonates **9**, **12** and La(tmhd)_3_ (He, 10 ^o^/min).

TG-DTA results for homometallic **9** and **11** are compared to La(tmhd)_3_ in Figure 6. Judging by the amount of nonvolatile residue, new homometallic complexes decompose when heated at atmospheric pressure.

### 2.3. Magnetic Properties

**Figure 7:**
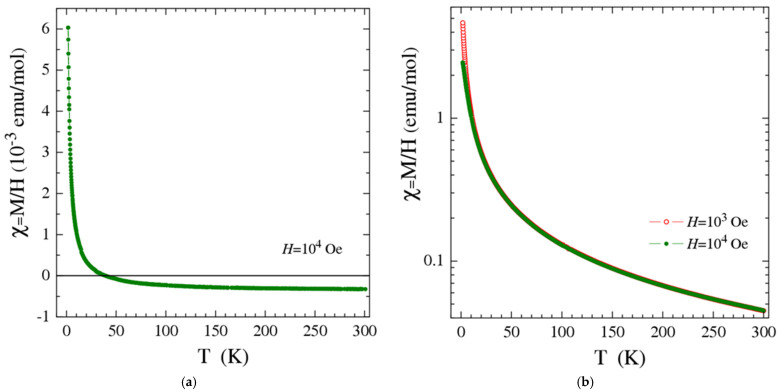
Temperature dependences of the magnetic susceptibility *χ* measured for the **12** (**a**) and **11** (**b**) complexes at the magnetic fields *H* = 1; 10 kOe.

**Figure 8:**
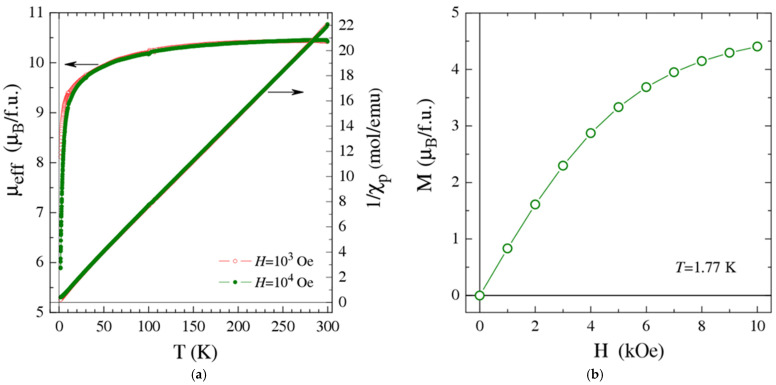
(**a**) Temperature dependences of *μ*_eff_ and 1/*χ*_p_ measured for the Dy(L^3^)_3_ (**11**) complex at the magnetic fields *H* = 1; 10 kOe. The depicted effective moment *μ*_eff_ is calculated for the case of non-interacting magnetic moments (*θ* = 0). (**b**) Magnetic-field dependence of the magnetization measured for complex **11** at *T* = 1.77 K.

Among the studied complexes, the homometallic tris-diketonates **11** and **12** represent the simplest cases for discussing magnetic properties. As expected, **12** exhibits diamagnetic behavior owing to the non-magnetic (*J* = 0) state of Lu^3+^ ions, except for the low-temperature region (*T* < 30 K) where impurities of other rare-earth elements at the level of a few tenths of a percent provide a paramagnetic upturn of the magnetic susceptibility (Figure 7a). In its turn, **11** containing Dy^3+^ ions (*S* = 5/2, *L* = 5, *J* = 15/2) demonstrates paramagnetic behavior throughout the available temperature range of 1.77–300 K (Figure 7b). As can be seen in Figure 8a the 1/*χ*_p_(*T*) curve deviates slightly from linearity and hence *χ*_p_(*T*) deviates from the simple Curie–Weiss dependence $\chi_{p}(T)=N_{A}\mu_{\mathrm{eff}}^{2}/3k_{B}(T-\theta )$ (*N*_A_ and *k*_B_ are the Avogadro number and the Boltzmann constant) implying the effective magnetic moment *µ*_eff_ to be temperature dependent. Nevertheless, the Curie–Weiss fit can still be used in the lowest-temperature region to roughly estimate the Weiss constant *θ* that characterizes the interaction between Dy^3+^ ions. The evaluated *θ* has turned out to be less than 0.6 K in magnitude pointing to a very weak, if any, magnetic interaction between Dy^3+^ ions. The effective magnetic moment calculated for Dy(L^3^)_3_ (**11**) under the assumption of non-interacting magnetic moments (*θ* = 0) gradually changes from *µ*_eff_ ≈ 10.45 *μ*_B_ at *T* = 300 K down to *µ*_eff_ ≈ 8.1 *μ*_B_ at *T* = 1.77 K (Figure 8a). Apparently, the high-temperature *µ*_eff_ value is close to the theoretical effective moment of a free Dy^3+^ ion *µ*_eff_(Dy) ≈ 10.64 *μ*_B_, while the gradual decrease in *µ*_eff_ upon cooling is predominantly caused by the crystal-field splitting of the ground state ^6^H_15/2_ multiplet.

To clarify the magnetic state of the Dy^3+^ ions, one can examine the magnetic-field dependence of the magnetization that should follow the expression $M(H)=N_{A}g\mu_{B}JB_{J}(\frac{g\mu_{B}}{k_{B}T}JH)$, where *B_J_*(*x*) is the Brillouin function. The shape of the *M*(*H*) dependence measured at *T* = 1.77 K turns out to be close to that expected for free Dy^3+^ ions with *J* = 15/2 and *g* = 4/3 [18], but the magnitude of the magnetization does not fit these parameters being less than half of the expected (Figure 8b). Indeed, as can be seen in Figure 8b the magnetization tends to saturate at a level below 5 *μ*_B_ per Dy^3+^ ion while the expected value equals 10 *μ*_B_. This discrepancy can be naturally resolved by taking into account a strong anisotropy of the *g* factor that Dy^3+^ ions develop at low temperatures, especially when placed in a low-symmetry environment [18,19,20]. In the case of a strongly anisotropic *g* factor, the magnetization along the easy axis of Dy^3+^ ions mimics the behavior of free ions, while the transverse magnetization is drastically suppressed [19]. After averaging the crystallographic orientation for a powder sample, the behavior of *M*(*H*), shown in Figure 8b, is naturally obtained.

Having established that it is the crystal-field effects that cause *µ*_eff_ to decrease with cooling (Figure 8a), we can conclude that the actual Weiss constant is much less in magnitude than the apparent *θ* ≈ −0.6 K. Although the dipole–dipole mechanism that usually dominates the interaction of rare-earth Ln^3+^ ions is generally rather weak, a hypothetic dimer state of large-moment (*J* = 15/2) Dy^3+^ ions would exhibit a noticeably larger *θ* than the actual one; the small actual *θ* is indicative of a large distance between Dy^3+^ ions and speaks in favor of a monomer crystal structure of **11**.

All the studied heterometallic complexes despite the presence of Ln^3+^ dimers in their structure were found to behave as almost ideal paramagnets over the available temperature range of 1.77–300 K without any sign of significant inter-ionic magnetic interactions (a detailed description is given in Supplementary Materials). The Gd-Cu complex was the only case when a very weak yet detectable inter-ionic interaction manifested in the Weiss constant *θ* ≈ −0.4 K was observed (Figure S6). Given its small magnitude, the Gd^3+^ -Gd^3+^ interaction in the binuclear structural fragment is most likely governed by the dipole–dipole mechanism rather than the exchange one.

In the cases of La-, Pr-, and Sm-based heterometallic complexes, the absence of noticeable inter-ionic magnetic interactions was caused by one and the same reason—a small or zero magnetic moment of the Ln^3+^ ion at low temperatures. Indeed, the *µ*_eff_(*T*) data taken for the Pr-Cu complex (Figure S7) pointed to the singlet ground state of each Pr^3+^ ion—a state quite common for Pr^3+^ ions, for instance, in a hexagonal or cubic environment [21]. The data obtained for the Sm-Cu complex implied the doublet ±1/2 ground state of Sm^3+^ (*J* = 5/2) ions which is weakly magnetic due to the small *g* factor (Figure S8). In the available temperature range down to 1.77 K, all the inter-ionic magnetic interactions are much weaker than the thermal energy *k*_B_*T*. Hence, from the point of view of magnetic properties, all the studied heterometallic complexes can be considered as a set of virtually non-interacting ions; all the observed magnetic peculiarities, if any, are associated exceptionally with specific properties of single ions in a particular structural environment. In this respect, the effective magnetic moments *µ*_eff_ of Pr- and Sm-based complexes were found to be small at low temperatures but increase considerably upon heating (up to *µ*_eff_(300 K) ≈ 5.38 *μ*_B_ and 2.87 *μ*_B_, respectively) owing to the excited levels of the Pr^3+^ and Sm^3+^ multiplets. The same is true for magnetic transition metal ions present in the studied heterometallic complexes. No sign of inter-ionic magnetic interactions was found for transition metal ions as well, yet the effective magnetic moments of Co^2+^ (*S* = 3/2) and Ni^2+^ (*S* = 1) changed significantly with temperature (Figures S9 and S10) due mostly to the zero-field spitting of their multiplets by the crystal-field [22].

## 3. Experimental

### 3.1. Synthesis

TML_2_ complexes with L^1^ and L^2^ were courtesy of IOS UB RAS (www.iosuran.ru, accessed on 18 October 2022). The general procedures for the preparation of TML_2_ have been described before [10,12,23,24,25]. For Ln(tmhd)_3_, the general method was used [17]. For the synthesis of heterometallic complexes, 200 mg of Ln(tmhd)_3_ (Ln = La, Pr, Sm, Gd) dissolved in 10 mL of toluene was mixed with an equimolar amount of TML_2_ (L = L^1^ or L^2^; TM = Cu, Co, Ni, Mn) dissolved in 10 mL of chloroform in a small cylindrical vessel covered with a paper cap and put into a small chamber with dry nitrogen flow. The cell was placed under a flow of dry nitrogen. When the volume of the solution was halved, an equal volume of heptane was added. As the solvent dries, a two-phase mixture of crystals is formed. The biphasic mixture was separated by vacuum sublimation or extraction (for Cu and Mn). For sublimation, the dried mixture was placed in a quartz boat and heated in a gradient tube furnace at T = 130 °C (P = 10^−2^ Torr). A volatile TM complex was condensed at the walls of the glass tube. A nonvolatile residue was recrystallized from toluene giving crystals of a heterometallic complex. Cu or Mn diketonate well soluble in heptane was extracted from the reaction products mixture in the following way. After the addition of heptane, the mixture was filtered, and a residue was washed several times with small portions of heptane. TM diketonate was collected after removing the solvent. The residue from the filter was recrystallized from toluene giving crystals of the heterometallic complex. The composition of the obtained crystals was determined using elemental analysis and XRD. As a result, we have identified and characterized a series of new heterometallic complexes on the basis of dimethoxy-substituted β-diketonates [(La(L^1^)_2_tmhd)_2_Cu(tmhd)_2_] (**1**), [(Pr(L^1^)_2_tmhd)_2_Cu(tmhd)_2_] (**2**), [(Sm(L^1^)_2_tmhd)_2_Cu(tmhd)_2_] (**3**), [(Gd(L^1^)_2_tmhd)_2_Cu(tmhd)_2_] (**4**), [(La(L^1^)_2_tmhd)_2_Co(tmhd)_2_] (**5**), [(La(L^1^)_2_tmhd)_2_Ni(tmhd)_2_] (**6**), [(La(L^1^)_2_tmhd)_2_Mn(tmhd)_2_] (**7**), [(La(L_2_)_2_tmhd)_2_Cu(tmhd)_2_] (**8**). **1**-[(La(L^1^)_2_tmhd)_2_Cu(tmhd)_2_] Anal. Calc. for C_72_H_108_O_24_F_12_CuLa_2_, **(**%): C, 44.9; H, 5.7; F, 11.8. Found: C, 44.2; H, 5.7; F, 12.1. MP(DTA) 216 °C. **2**–[(Pr(L^1^)_2_tmhd)_2_Cu(tmhd)_2_] Anal. Calc. for C_72_H_108_O_24_F_12_CuPr_2_, (%): C, 44.8; H, 5.6; F, 11.8. Found: C, 44.5; H, 5.3; F, 11.2. MP(DTA) 201 °C. **3**–[(Sm(L^1^)_2_tmhd)_2_Cu(tmhd)_2_] Anal. Calc. for C_72_H_108_O_24_F_12_CuSm_2_, (%): C, 44.8; H, 5.5; F, 11.7. Found: C, 44.1; H, 5.5; F, 11.5. MP(Kofler plate) 170 °C. **4**-[(Gd(L^1^)_2_tmhd)_2_Cu(tmhd)_2_] Anal. Calc. for C_72_H_108_O_24_F_12_CuGd_2_, (%):C, 44.1; H, 5.5; F, 11.6. Found: C, 44.0; H, 5.4; F, 11.4. MP(Kofler plate) 138 °C. **5**-[(La(L^1^)_2_tmhd)_2_Co(tmhd)_2_] Anal. Calc. for C_72_H_108_O_24_F_12_CoLa_2_, (%): C, 45.0; H, 5.6; F, 11.9. Found: C, 44.9; H, 5.7; F, 11.8; MP(Kofler plate) 185 °C. **6**-[(La(L^1^)_2_tmhd)_2_Ni(tmhd)_2_] Anal. Calc. for C_72_H_108_O_24_F_12_NiLa_2_, (%): C, 45.0; H, 5.6; F, 11.9. Found: C, 44.9; H, 5.7; F, 11.8; MP(Kofler plate) 178 °C. **8**-[(La(L_2_)_2_tmhd)_2_Cu(tmhd)_2_] Anal. Calc. for C_76_H_116_O_24_F_12_CuLa_2_, (%): C, 46.1; H, 5.9; F, 11.5. Found: C, 46.4; H, 5.7; F, 11.5; MP(Kofler plate) 225 °C.

New monometallic complexes [La(L^3^)_3_]_2_ (**9**), Tb(L^3^)_3_ (**10**), Dy(L^3^)_3_ (**11**), Lu(L^3^)_3_ (**12**)**,** were obtained by the same technique using a molar ratio of Ln(tmhd)_3_ to TM(L^3^)_2_ equal to 2:3. The obtained compounds are colorless solids; only **9** is crystalline. Anal. Calc. for C_24_H_30_O_9_F_9_La (**9**), (%):C, 37.3; H, 3.9; F, 22.1. Found: C, 36.5; H, 4.1; F, 22.5. ^1^H NMR (500 MHz, CDCl_3_, 25 °C): δ 6.10 (2H, w, γ-CH, peripheral), 5.51 (4H, w, γ-CH, internal), 3.61 (6H, w, -OCH_3_, peripheral), 3.20 (12H, w, -OCH_3_, internal), 1.31 (36H, s, -(CH3)2) ^19^F NMR (470 MHz, C_6_D_6_, 25 °C, CClF_3_ as reference): δ 75.78 and 76.22 in 1:2 ratio for internal and peripheral -CF_3_ groups. MP (Kofler plate/DTA) 225–229/224 °C. Anal. Calc. for C24H30O9F9Tb (**10**) (%):C, 36.4; H, 3.8; F, 21.6. Found: C, 35.6; H, 4.0; F, 21.5. Anal. Calc. for C24H30O9F9Dy (**11**) (%):C, 36.2; H, 3.8; F, 21.5. Found: C, 35.4; H, 4.1; F, 21.4. Anal. Calc. for C24H30O9F9Lu (**12**) (%):C, 35.7; H, 3.7; F, 21.2. Found: C, 35.5; H, 4.1; F, 21.5. ^1^H NMR (500 MHz, CDCl_3_, 25 °C): δ 6.3 (3H, w, γ-CH), 3.18 (9H, s, -OCH3), 1.19 (18H, s, -(CH_3_)_2_) ^19^F NMR (470 MHz, C_6_D_6_, 25 °C): δ 87.5–90.5 multiplets.

### 3.2. Physical Measurements

The elemental microanalysis (C, H, N) was performed with a Euro EA 3000 elemental analyzer; the content of fluorine was carried out at the laboratory of microanalysis of NIOCH SB RAS (http://web.nioch.nsc.ru/nioch/en/institute-nav/the-centers-of-collective-use accessed on 18 October 2022). To quickly verify the occurrence of both Ln and TM in the same phase, X-ray fluorescence (XRF) with a Bruker M1 Mistral was used. NMR was recorded with a Bruker Avance III/500. The thermal properties of the compounds in the condensed phase were investigated using thermogravimetric and differential thermal analyses (TG-DTA) on a TG 209 F1 Iris (NETZSCH) thermobalance in the standard open corundum crucible. The measurements were performed under atmospheric pressure in helium flow (30–40 cm^3^ min^−1^) with a heating rate of 10 °C × min^−1^ within the temperature range 25–500 °C. The sample weight was ~10 mg. For the XRD study of the solid residue after the compound thermolysis, a ~200 mg sample decomposed in a tube furnace at 600 °C in air.

Single crystals used to determine the structure of **1**, **2**, **5**, **6**, **8**, and **9** were obtained directly from their solutions after slow evaporation of the solvent (toluene). The unit cell parameters and experimental intensities to solve the crystal structure were measured with a Bruker Kappa Apex II CCD diffractometer (MoKα radiation and graphite monochromator) using *φ, ω* scans of narrow (0.5°) frames. The structures of **1** and **2** were solved by direct methods by the *SHELXS-97* program [26] and **5**, **6**, **8**, and **9** by *SHELXT* 2014/5 [27]. **1** and **2** were refined in an anisotropic (isotropic for H and F in a minor part of disordered CF_3_ groups) approximation using the *SHELXL-2014* program [27] and **5**, **6**, **8**, **9**—with *SHELXL2018*/3 [28]. The hydrogens’ positions were calculated with the riding model. The CF_3_ groups of both compounds **1** and **2** are disordered due to rotation approximately as 9:1 and ^t^Bu(C25) group–as 6:4. The ^t^Bu groups of tmhd in **5** are disordered in approximate ratios 3:1 and 3:2, and CF_3_ groups of L^1^ in ratios 9:1 and 3:2. In **8**, methyl and methoxy groups of L^2^ are disordered in ratio 1:1 for noncoordinated and 3:1 for coordinated methoxy-group. One of ^t^Bu group of tmhd in **8** is also disordered due to rotation in ratio 3:2. Compounds **1**, **2** and **5**, **6**, **8** demonstrate the same space group, crystal packing and close geometry of the ligands. Asymmetry unit of **9** includes two independent molecules one of which demonstrate disordered due to rotation methoxy-group of L^1^ in ratio 3:2. Another one has disordered CF_3_ groups in ratio 9:1. Crystallographic data are summarized in Table S1. CCDC deposition numbers: 1534092 for **1**, 1534091 for **2**, 2175224 for **5**, 2175225 for **6**, 2175226 for **8**, 2175227 for **9**. These data can be obtained free of charge via http://www.ccdc.cam.ac.uk/conts/retrieving.html, accessed on 18 October 2022 (or from the CCDC, 12 Union Road, Cambridge CB2 1 EZ, UK; Fax: +44-12-2333-6033; E-mail: deposit@ccdc.cam.ac.uk).

The X-ray powder diffraction (XRD) was carried out using a Shimadzu XRD-7000 diffractometer (CuKα radiation, Ni filter, 5 to 50° 2θ angle range and a step 0.03° 2θ) at room temperature and atmospheric pressure. The products were ground in an agate mortar; the powder was deposited onto the polished side of the standard quartz cuvette. A polycrystalline silicon sample prepared similarly was used as the external standard. Indexing of the diffraction patterns was carried out using data for compounds reported in the PDF-2 database [29] and by the results of the single crystal study.

Magnetic properties of polycrystalline samples were studied using a Quantum Design MPMS-XL SQUID magnetometer in the temperature range *T* = 1.77–300 K at magnetic fields *H* = 0–10 kOe. To determine the paramagnetic component of the molar magnetic susceptibility *χ*_p_(*T*), the temperature-independent diamagnetic contribution *χ*_d_ and a possible contribution of ferromagnetic micro impurities χ_F_ were subtracted from the measured values of the total molar susceptibility *χ* = *M/H*: *χ*_p_(*T,H*) = *χ*(*T,H*)–*χ*_d_–*χ*_F_(*T,H*). The value of *χ*_d_ was calculated according to the additive Pascal scheme. In its turn the ferromagnetic contribution *χ*_F_, if any, was evaluated from the field dependences of the magnetization *M*(*H*) by decomposing the latter into a part changing linearly with a magnetic field (associated with diamagnetic and paramagnetic contributions), and a ferromagnetic part tending to saturate at magnetic fields of a few kOe.

## 4. Conclusions

This study has shown that the use of fluorinated methoxy-substituted diketones for the synthesis of volatile heterometallic complexes containing lanthanides and typical transition metals still requires a further search for methods and approaches to achieve the desired result. It has been found so far for these systems that during the cocrystallization of monometallic complexes there is the exchange of ligands by redistributing the latter following the principle of concentrating fluorine-containing ligands around lanthanide cations. Apparently, this is due to the fact that the electronegativity of lanthanides is lower than that of transition metals, and they tend to form complexes with a more polar coordination bond. In addition, large coordination numbers lead to the fact that the available methoxy groups are also coordinated by lanthanides without alternatives so that a homometallic aggregate is formed rather than a heterometallic one. The peculiarity of lanthanides is also manifested in that the set of products split into two series for La-Gd and Tb-Lu, which often occurs in their coordination chemistry. In particular, the former tend to form dimers in contrast to the latter. For dimethoxy diketonates L^1^ and L^2^ heterometallic complexes are formed for Ln = La-Gd, but not for Ln = Tb-Lu. For mono-methoxy diketonate L^3^ all Ln under discussion give no heterometallic products. At the same time, the effect of steric factors cannot be excluded, because even the presence of free methoxy groups does not guarantee the formation of heterometallic complexes as, e.g., for [La(L^3^)_3_]_2_ (**9**) or Lu(L^3^)_3_ (**12**) in contrast to [Ln(L^1^)_2_tmhd]_2_. In addition, the more rigid structure of TM complexes also does not allow them to adjust to it. Nevertheless, a number of unique heterocomplexes with interesting structures were obtained, which will be useful for the further design of polynuclear compounds with the required properties.

In addition, a reliable way to synthesize anhydrous lanthanide complexes with fluorinated diketonates has been found.

## Figures and Tables

**Table 1:** Comparison of geometry parameters for [(Ln(L^1^)_2_tmhd)_2_TM(tmhd)_2_], Ln = La, Pr, M= Cu, Ni, Co.

#-Ln	<Ln-O_ch_>	<Ln-O_chb_>	Ln-OCH_3_ *	Ln_Ln	TM	TM-O_ch_	TM-OCH_3_	<O_ch_-Ln-O_chb_>	O_ch_-TM-O_ch_
**1-La**	2.46(8)	2.63(2)	2.765(3) 2.759(3)	3.854(3)	Cu	1.915(3) 1.941(3)	2.604(3)	65.4(4)	92.4(1)
**2-Pr**	2.42(8)	2.59(1)	2.752(3) 2.739(3)	3.793(3)	Cu	1.915(3) 1.941(3)	2.602(3)	66.2(4)	92.5(1)
**5-La**	2.50(8)	2.63(2)	2.767(3) 2.747(3)	3.834(3)	Co	1.975(3) 2.009(3)	2.370(3)	65.3(4)	70.3(1)
**6-La**	2.50(8)	2.63(2)	2.769(3) 2.753(3)	3.838(3)	Ni	1.956(3) 1.992(3)	2.306(3)	65.2(4)	70.5(1)
**8-La**	2.47(8)	2.65(2)	2.774(3) 2.803(3)	3.838(3)	Cu	1.910(3) 1.928(3)	2.685(3)	65.3(4)	92.4(1)

Bonds: ch—chelated; chb—chelato-bridged; *—first value is for L where both methoxy groups are coordinated. #—index number of the compound.

**Table 2:** Selected geometry parameters for **9**.

Bond Length, Å			Angle, °
La2-O41	2.504(3)	O41-La2-O42	66.9(9)
La2-O42	2.613(3), 2.612 *	La2-O42-La2	93.1(9)
La2-O43	2.704(3)	O42-La2-O43	58.9(9)
La2-O51	2.505(3)	O51-La2-O52	65.7(9)
La2-O52	2.627(3), 2.622 *	La2-O52-La2	92.6(9)
La2-O53	2.702(3)	O52-La2-O53	56.1(9)
La2-O61	2.490(3)	O61-La2-O62	69.6(9)
La2-O62	2.454(3)		

* bridging distance.

## Data Availability

Not applicable.

## Supplementary Materials

The following supporting information can be downloaded at: https://www.mdpi.com/article/10.3390/molecules27238400/s1, Table S1: XRD data for complexes **1**, **2**, **5**, **6**, **8**, **9**; Figure S1: A view of the cell packing along Z axis in crystals of **2**; Figure S2: XRD data for solid residue after sintering [(La(L^1^)_2_tmhd)_2_Cu(tmhd)_2_] in comparison with appropriate PDF files; Figure S3: XRD data for solid residue after sintering [(La(L^1^)_2_tmhd)_2_Co(tmhd)_2_] in comparison with appropriate PDF files; Figure S4: XRD data for solid residue after sintering [(La(L^1^)_2_tmhd)_2_Ni(tmhd)_2_] in comparison with appropriate PDF files; Figure S5: XRD data for solid residue after sintering [(La(L^1^)_2_tmhd)_2_Mn(tmhd)_2_] in comparison with appropriate PDF files; Figure S6: (a) Temperature dependence of the magnetic susceptibility χ measured for the Gd-Cu complex at the magnetic-field H = 1 kOe. (b) Temperature dependences of *μ*_eff_ and 1/χp. The depicted effective moment *μ*_eff_ is calculated for the case of non-interacting magnetic moments (θ = 0). Pr-Cu complex (**2**); Figure S7: (a) Temperature dependence of the magnetic susceptibility χ measured for the Pr-Cu complex at the magnetic fields H = 1; 10 kOe. (b) Temperature dependences of *μ*_eff_ and 1/χp. The depicted effective moment *μ*_eff_ is calculated for the case of non-interacting magnetic moments (θ = 0). Sm-Cu complex (**3**); Figure S8: (a) Temperature dependence of the magnetic susceptibility χ measured for the Sm-Cu complex at the magnetic fields H = 1; 10 kOe. (b) Temperature dependences of *μ*_eff_ and 1/χp. The depicted effective moment *μ*_eff_ is calculated for the case of non-interacting magnetic moments (θ = 0). La-Co complex (**5**); Figure S9: (a) Temperature dependence of the magnetic susceptibility χ measured for the La-Co complex at the magnetic fields H = 1; 10 kOe. (b) Temperature dependences of *μ*_eff_ and 1/χp. The depicted effective moment *μ*_eff_ is calculated for the case of non-interacting magnetic moments (θ = 0). (**b**) Magnetic-field dependence of the magnetization measured at T = 1.77 K. La-Ni complex (**6**); Figure S10: (a) Temperature dependence of the magnetic susceptibility χ measured for the La-Ni complex at the magnetic fields H = 1; 10 kOe. (b) Temperature dependences of *μ*_eff_ and 1/χp. The depicted effective moment *μ*_eff_ is calculated for the case of non-interacting magnetic moments (θ = 0).

## Sample Availability

Samples of the compounds are not available from the authors.
