# Supplementary material for: Structure and Properties of Heterometallics Based on Lanthanides and Transition Metals with Methoxy-β-Diketonates
Source: Molecules. 2022 Dec 1;27(23):8400. doi: 10.3390/molecules27238400 (PMC9736739; doi:10.3390/molecules27238400)
Supplement: Supplementary file 1 [file molecules-27-08400-s001.zip › molecules-2012516-supplementary.pdf]

Supplementary Materials to paper

Structure and properties of heterometallics based on lanthanides and transition metals with methoxy-β-diketonates

Vladislav V. Krisyuk[1], Samara Urkasym kyzy[1], Tatyana V. Rybalova[2], Ilya V. Korolkov[1], Mariya A. Grebenkina[1,3] and Alexander .N. Lavrov[1*]

[1] Nikolaev Institute of Inorganic Chemistry, SB RAS, Lavrentiev Ave. 3, 630090 Novosibirsk, Russia; kvv@niic.nsc.ru (V.V.K.)
[2] Vorozhtsov Novosibirsk Institute of Organic Chemistry SB RAS, Lavrentiev Ave. 9, 630090 Novosibirsk, Russia
[3] Novosibirsk National Research State University, 1 Pirogova Str., 630090 Novosibirsk, Russia
* Correspondence: lavrov@niic.nsc.ru (A.N.L.), kvv@niic.nsc.ru (V.V.K.)

Content

Table S1: XRD data for complexes **1,2,5,6,8,9**.
Figure S1: A view of the cell packing along Z axis in crystals of **2**.
Figure S2: XRD data for solid residue after sintering [(La(L^1)$_2$tmhd)$_2$Cu(tmhd)$_2$] in comparison with appropriate PDF files.
Figure S3: XRD data for solid residue after sintering [(La(L^1)$_2$tmhd)$_2$Co(tmhd)$_2$] in comparison with appropriate PDF files.
Figure S4: XRD data for solid residue after sintering [(La(L^1)$_2$tmhd)$_2$Ni(tmhd)$_2$] in comparison with appropriate PDF files.
Figure S5: XRD data for solid residue after sintering [(La(L^1)$_2$tmhd)$_2$Mn(tmhd)$_2$] in comparison with appropriate PDF files.

Results of magnetic measurements for the heterocomplexes

Gd-Cu complex (**4**)
Figure S6: (a) Temperature dependence of the magnetic susceptibility χ measured for the Gd-Cu complex at the magnetic field H=1 kOe. (b) Temperature dependences of μ_{eff} and 1/χp. The depicted effective moment μ_{eff} is calculated for the case of non-interacting magnetic moments (θ = 0).

Pr-Cu complex (**2**)
Figure S7: (a) Temperature dependence of the magnetic susceptibility χ measured for the Pr-Cu complex at the magnetic fields H = 1; 10 kOe. (b) Temperature dependences of μ_{eff} and 1/χp. The depicted effective moment μ_{eff} is calculated for the case of non-interacting magnetic moments (θ = 0).

Sm-Cu complex (**3**)
Figure S8: (a) Temperature dependence of the magnetic susceptibility χ measured for the Sm-Cu complex at the magnetic fields H = 1; 10 kOe. (b) Temperature dependences of μ_{eff} and 1/χp. The depicted effective moment μ_{eff} is calculated for the case of non-interacting magnetic moments (θ = 0).

La-Co complex (**5**)
Figure S9: (a) Temperature dependence of the magnetic susceptibility χ measured for the La-Co complex at the magnetic fields H = 1; 10 kOe. (b) Temperature dependences of μ_{eff} and 1/χp. The depicted effective moment μ_{eff} is calculated for the case of non-interacting magnetic moments (θ = 0). (c) Magnetic field dependence of the magnetization measured at T = 1.77 K.

La-Ni complex (**6**)
Figure S10: (a) Temperature dependence of the magnetic susceptibility χ measured for the La-Ni complex at the magnetic fields H = 1; 10 kOe. (b) Temperature dependences of μ_{eff} and 1/χp. The depicted effective moment μ_{eff} is calculated for the case of non-interacting magnetic moments (θ = 0).

Table S1: XRD data for complexes **1,2,5,6,8,9.**

Compound	**1**	**2**	**5**	**6**	**8**	**9**
Empirical formula	$C_{72}H_{108}F_{12}O_{24}CuLa_2$	$C_{72}H_{108}F_{12}O_{24}CuPr_2$	$C_{72}H_{108}CoF_{12}La_2O_{24}$	$C_{72}H_{108}F_{12}La_2NiO_{24}$	$C_{76}H_{116}CuF_{12}La_2O_{24}$	$C_{48}H_{60}F_{18}La_2O_{18}$
Formula weight	1926.94	1930.95	1922.33	1922.11	1983.04	1544.78
	200(2) K	200(2) K	150(2) K	150(2) K	150(2) K	110(2) K
Wavelength Å	0.71073	0.71073	0.71073	0.71073	0.71073	0.71073
Crystal system	Monoclinic	Monoclinic	Monoclinic	Monoclinic	Monoclinic	Monoclinic
Space group	P2(1)/n	P2(1)/n	$P2_1/n$	$P2_1/n$	$P2_1/n$	$P2_1/c$
Unit cell dimensions *a*, Å	13.0379(8)	13.0009(6)	13.0328(4)	13.0158 (7)	12.8652(5)	18.2778(4)
b, Å	21.985(1)	21.9732(1)	21.964(1)	21.993(2)	22.5354(9)	19.9517(3)
c, Å	15.0964(8)	15.0612(6)	14.6846(6)	14.5878(8)	15.2704(6	16.7143(3)
α,°	90	90	90	90	90	90
β,°	97.289(2)	97.354(2)	96.803 (1)	96.719 (2)	95.940 (2)	97.888 (1)
γ,°	90	90	90	90	90	90
Volume, $Å^3$	4292.1(4)	4267.2(3)	4173.8 (3)	4147.2 (4)	4403.5 (3)	6037.58 (19)
Z	2	2	2	2	2	4
Density (calcd.), $Mg.m^{-3}$	1.491	1.503	1.530	1.539	1.496	1.699
Abs. coefficient, mm^{-1}	1.317	1.466	1.30	1.33	1.29	1.52
F(000)	1966	1974	1962	1964	2030	3072
Crystal size, mm^3	0.32 x 0.08 x 0.03	0.57 x 0.11 x 0.05	0.25 × 0.08 × 0.04	0.26 × 0.22 × 0.05	0.12 × 0.1 × 0.09	0.29 × 0.16 × 0.14
Θ range for data collection, °	2.7 – 29.4	2.3 – 34.4	2.3 - 25.8	1.8 - 27.1	2.2 - 31.6	1.5 - 29.6
Index ranges	$-18 \le h \le 18$, $-30 \le k \le 30$, $-20 \le l \le 20$	$-20 \le h \le 20$, $-34 \le k \le 34$, $-23 \le l \le 22$	$-11 \le h \le 15$, $-17 \le k \le 26$, $-17 \le l \le 17$	$-16 \le h \le 12$, $-26 \le k \le 28$, $-18 \le l \le 18$	$-18 \le h \le 18$, $-33 \le k \le 33$, $-22 \le l \le 22$	$-22 \le h \le 25$, $-24 \le k \le 27$, $-23 \le l \le 20$

Reflections collected	94240	115128	20278	34638	209635	49738
Independent reflections	11842 [R(int) 0.049]	17896 [R(int) 0.047]	7937 [R(int) 0.028]	9149 [R(int) 0.159	14670 [R(int) 0.057	16948 [R(int) 0.044
Reflections $I > 2\sigma(I)$	9599	14502	6584	6009	12323	12912
Completeness to θ %	99.7, θ = 25.25°	99.9, θ = 25.25°	99.5, θ = 25.25°	100, θ = 25.25°	99.9, θ = 25.25°	99.8, θ = 25.25°
parameters / restraints	572/ 30	584/ 23	547 / 24	518 / 0	620 /79	834 / 6
Max. and min. transmission	0.678, 0.962	0.489, 0.930	0.615, 0.745	0.651, 0.746	0.679, 0.746	0.664, 0.746
Refinement method	Full matrix LSM against F^2	Full matrix LSM against F^2	Full matrix LSM against F^2	Full matrix LSM against F^2	Full matrix LSM against F^2	Full matrix LSM against F^2
Goodness-of-fit on *L4*	1.068	1.294	1.02	0.98	1.07	1.01
Final R indices $I > 2\sigma(I)$: R_1, wR_2	0.0333, 0.0857	0.0491, 0.1249	0.038, 0.1069	0.039, 0.085	0.046, 0.113	0.035, 0.064
Final R indices (all data): R_1, wR_2	0.0511, 0.1003	0.0696, 0.1368	0.0511, 0.115	0.1016, 0.097	0.057, 0.120	0.056, 0.072
Largest diff. peak / hole, e.Å^{-3}	1.03 / -0.87	2.29 / -1.02	0.83, -0.68	1.31, -1.54	1.60, -1.33	0.68, -0.69
*CCDC deposition numbers	1534092	1534091	2175224	2175225	2175226	2175227

* Cif-files can be obtained free of charge from The Cambridge Crystallographic Data Centre via www.ccdc.cam.ac.uk/data_request/cif

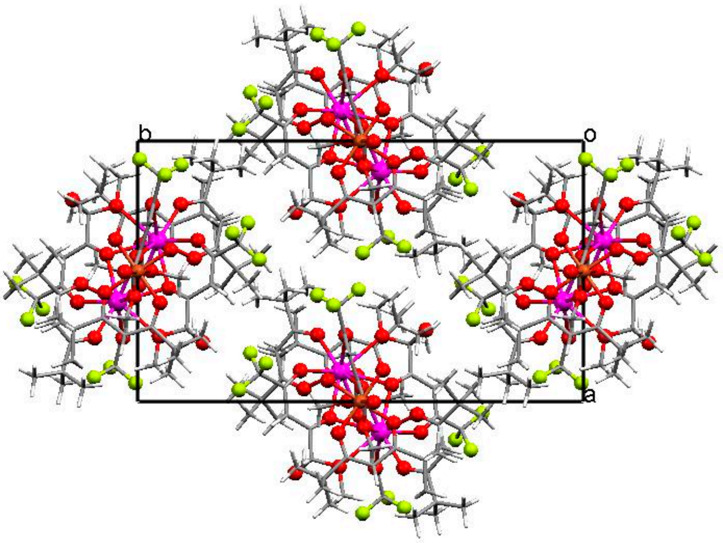

Figure S1: A view of the cell packing along *Z* axis in crystals of **2**.

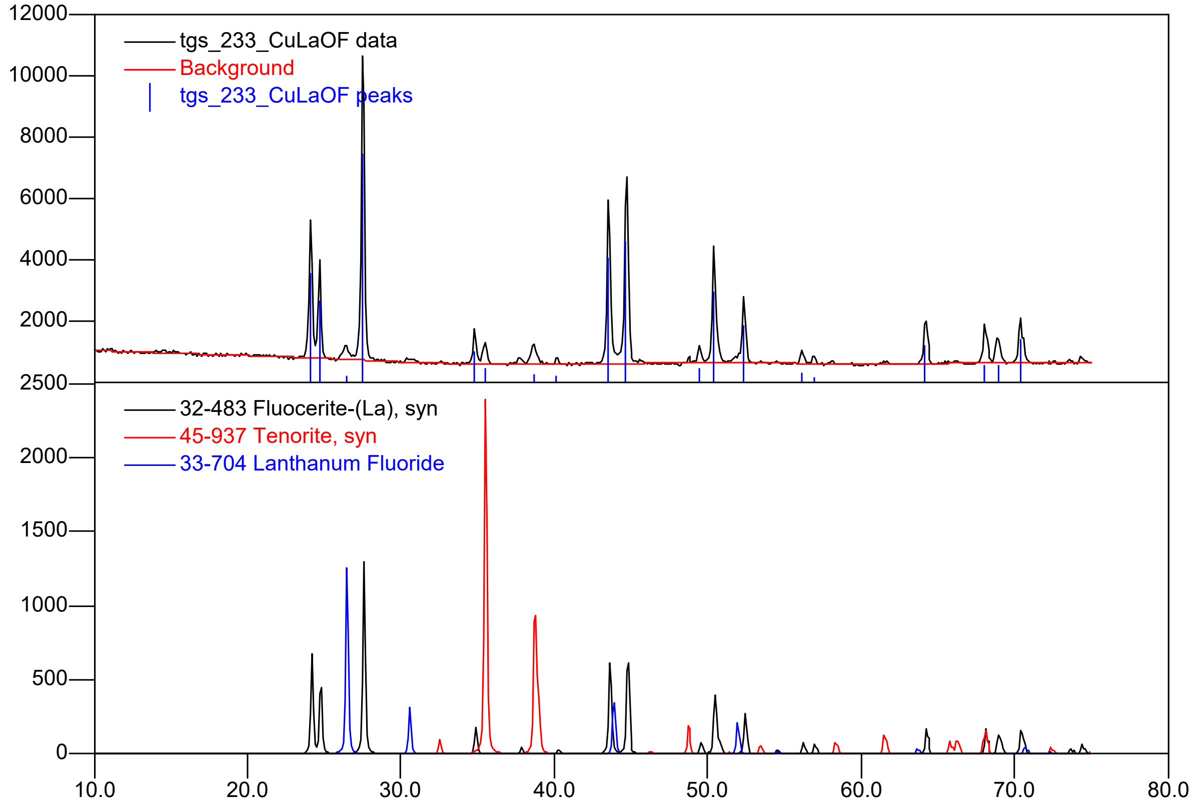

Figure S2: XRD data for solid residue after sintering [(La(L^1)$_2$tmhd)$_2$Cu(tmhd)$_2$] in comparison with appropriate PDF files.

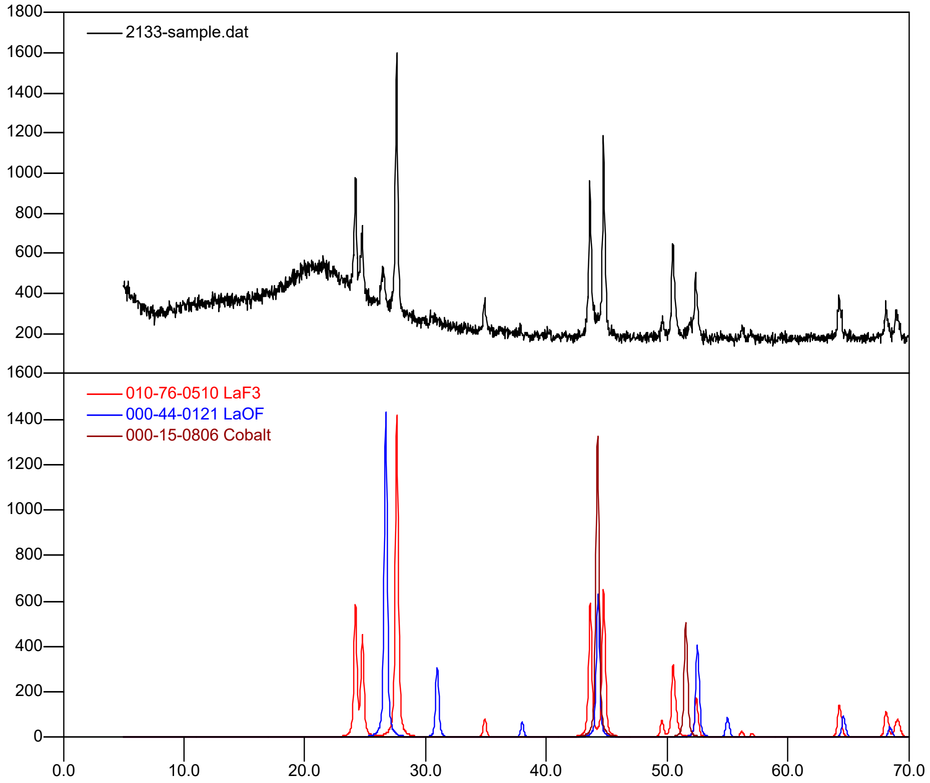

Figure S3: XRD data for solid residue after sintering $[(La(L^1)_2tmhd)_2Co(tmhd)_2]$ in comparison with appropriate PDF files

.

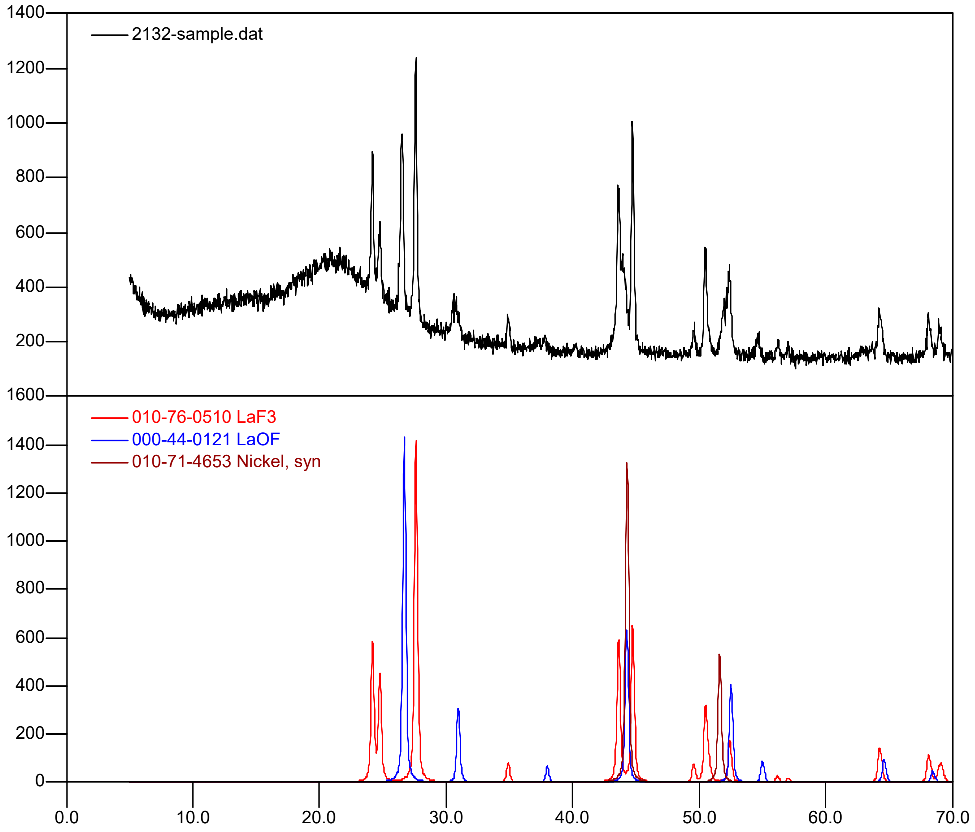

Figure S4: XRD data for solid residue after sintering $[(La(L^1)_2tmhd)_2Ni(tmhd)_2]$ in comparison with appropriate PDF files.

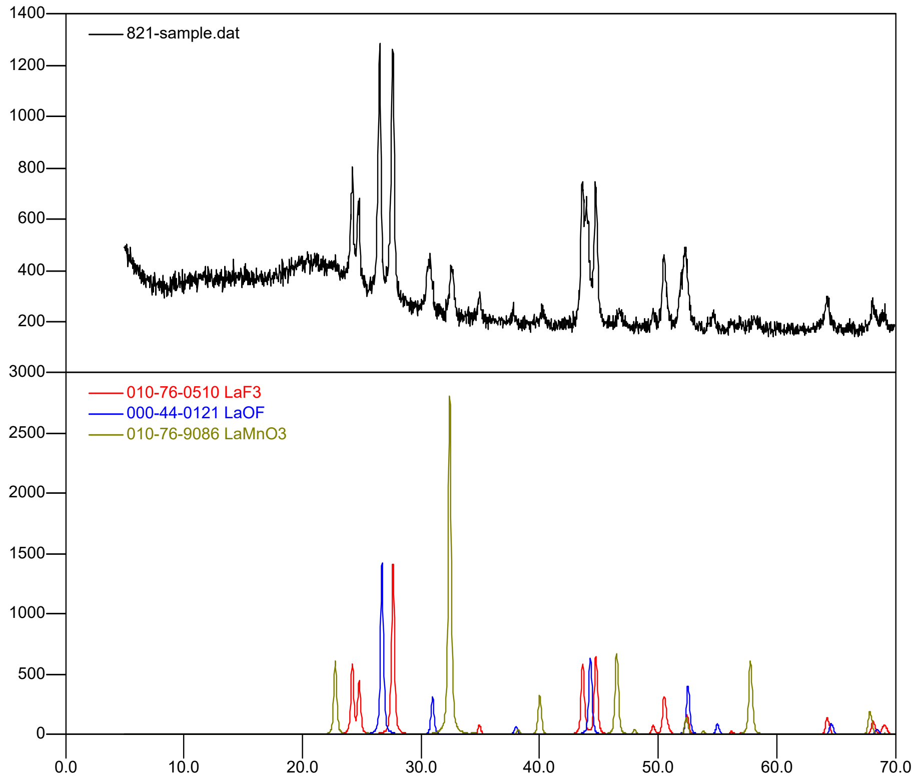

Figure S5: XRD data for solid residue after sintering [(La(L[1])$_2$tmhd)$_2$Mn(tmhd)$_2$] in comparison with appropriate PDF files.

Results of magnetic measurements for the heterocomplexes

Gd-Cu complex **(4)**

The Gd-Cu complex is found to exhibit paramagnetic behavior throughout the available temperature range without any magnetic phase transition (Figure S6a). The paramagnetic behavior can be well described by the Curie-Weiss dependence $\chi_p(T) = N_A\mu_{eff}^2/3k_B(T - \theta)$ with a nearly zero Weiss constant $\theta \approx -0.4$ K (Figure S6b). At high temperatures, the measured effective magnetic moment $\mu_{eff} \sim 11.47\ \mu_B$ corresponds very well to the values expected for two gadolinium ions Gd^{3+}, $\mu_{eff}(Gd) \approx 7.94\ \mu_B$, and one copper ion Cu^{2+}, $\mu_{eff}(Cu) \approx 2\ \mu_B$: $\mu_{eff} \approx \sqrt{2 \cdot 7.94^2 + 2^2} \approx 11.41\ \mu_B$. In the low-temperature region, the $1/\chi_p(T)$ dependence deviates from linearity and the calculated effective magnetic moment decreases slightly both due to the nonlinearity of the magnetization curve expected for Gd^{3+} ions (S = 7/2) in the region of fields $g\mu_B SH \sim k_B T$ and a very weak inter-ion exchange or dipole-dipole interactions. Thus, the studied compound can be considered as a set of virtually non-interacting gadolinium Gd^{3+} ions (S = 7/2) and Cu^{2+} ions (S = 1/2).

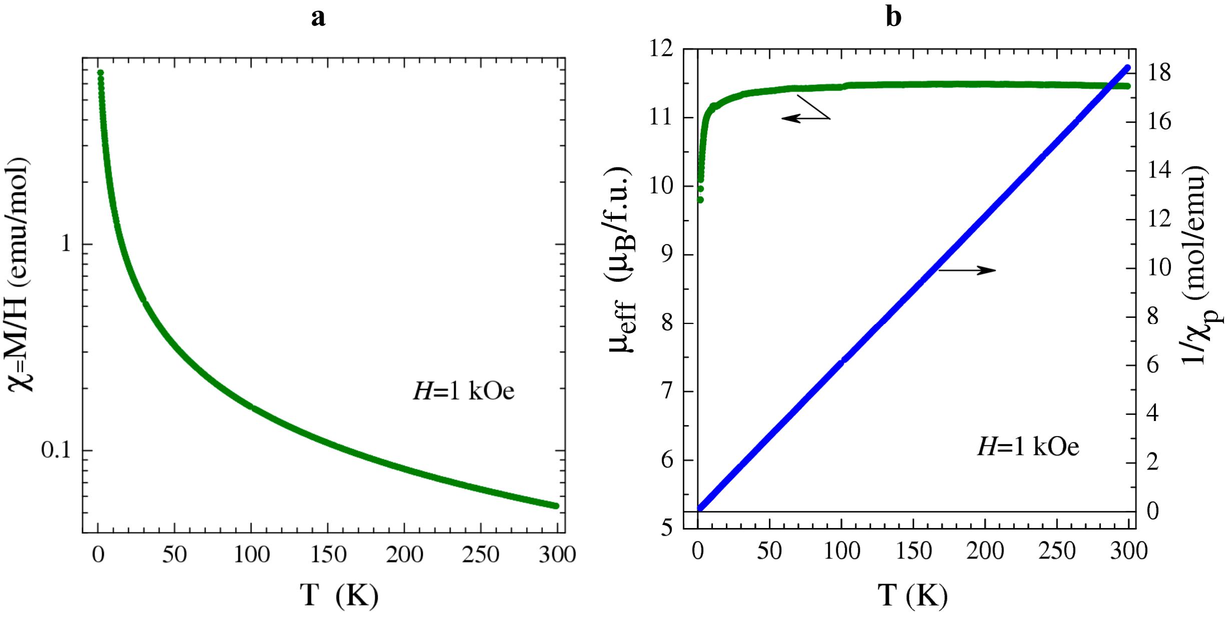

Figure S6: (a) Temperature dependence of the magnetic susceptibility χ measured for the Gd-Cu complex at the magnetic field H=1 kOe. (b) Temperature dependences of μ_{eff} and $1/\chi_p$. The depicted effective moment μ_{eff} is calculated for the case of non-interacting magnetic moments (θ = 0).

***Pr-Cu complex* (2)**

The Pr-Cu complex exhibits paramagnetic behavior throughout the available temperature range with a low-temperature peculiarity that at a first glance looks like a manifestation of inter-ion interactions (Figure S7a); on the $\chi(T)$ plot, it seems that the susceptibility begins to grow faster at $T < 7$-8 K. However, in fact it is the low-temperature region where the susceptibility behavior matches the Curie-Weiss dependence with the Weiss constant θ close to zero (Figure S7b), while at higher temperatures the susceptibility behavior is determined by the changes in effective moment $\mu_{\rm eff}$. In the highest temperature region of 200-300 K, the $\chi_{\rm p}(T)$ data can be described by the Curie-Weiss dependence with an effective magnetic moment $\mu_{\rm eff} \approx 5.38\ \mu_{\rm B}$ (Figure S2b), which agrees well with the value expected for two Pr^{3+} ions ($S = 1$, $L = 5$, $J = 4$) and one Cu^{2+} ion ($S = 1/2$, $g = 2.1$): $\mu_{\rm eff} \approx \sqrt{2\cdot 3.58^2 + 1.82^2} \approx 5.38\ \mu_{\rm B}$. On cooling, the effective magnetic moment decreases down to $\mu_{\rm eff} \approx 2.07\ \mu_{\rm B}$ at $T = 1.77$ K. This decrease corresponds in magnitude to the complete disappearance of the contribution from the praseodymium ions while the contribution from Cu^{2+} ions is kept intact. Since the disappearance of the magnetic contribution of Pr^{3+} ions as a result of their antiferromagnetic (AFM) interaction requires unrealistically strong exchange interactions, the most likely explanation is the singlet ground state of each Pr^{3+} ion, a state quite common for Pr^{3+} ions, for instance, in hexagonal or cubic environment [*1]. Consequently, at low temperatures the magnetic susceptibility of the Pr-Cu complex is predominantly determined by Cu^{2+} ions, while at higher temperatures the contribution from excited levels of the Pr^{3+} ion multiplet is added.

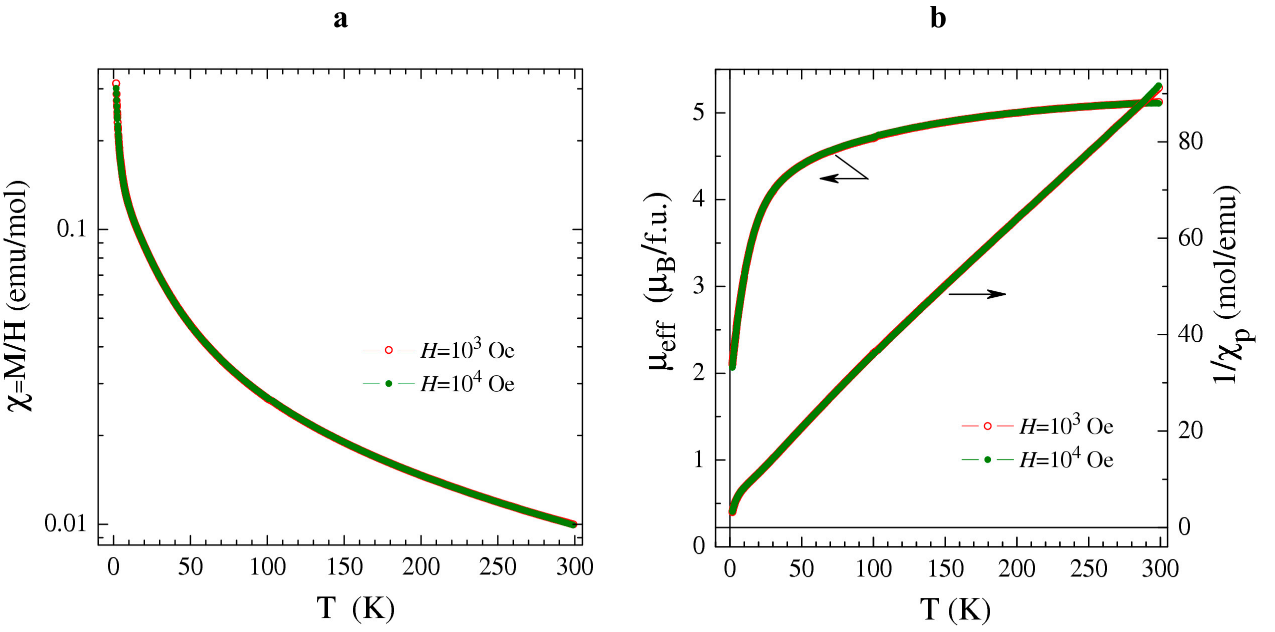

Figure S7: (a) Temperature dependence of the magnetic susceptibility χ measured for the Pr-Cu complex at the magnetic fields $H = 1$; 10 kOe. (b) Temperature dependences of $\mu_{\rm eff}$ and $1/\chi_{\rm p}$. The depicted effective moment $\mu_{\rm eff}$ is calculated for the case of non-interacting magnetic moments ($\theta = 0$).

Sm-Cu complex **(3)**

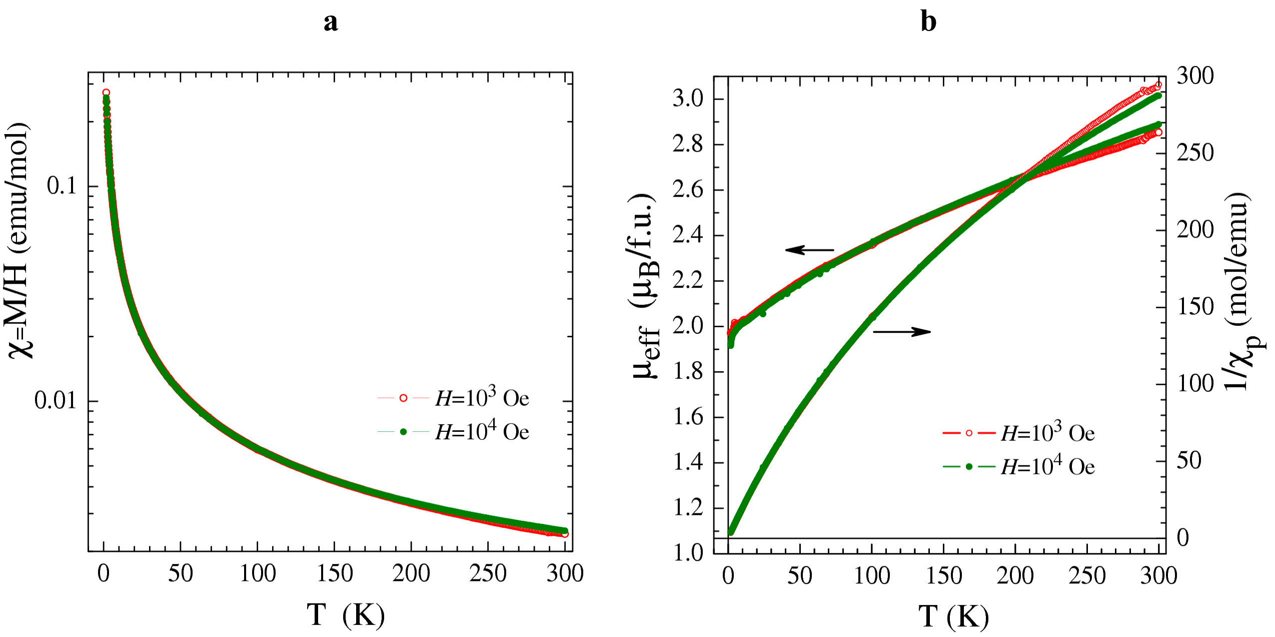

Figure S8: (a) Temperature dependence of the magnetic susceptibility χ measured for the Sm-Cu complex at the magnetic fields H = 1; 10 kOe. (b) Temperature dependences of μ_{eff} and $1/\chi_p$. The depicted effective moment μ_{eff} is calculated for the case of non-interacting magnetic moments ($\theta = 0$).

The Sm-Cu complex demonstrates paramagnetic behavior throughout the available temperature range (Figure S8a) without any noticeable sign of inter-ion interactions. The latter is confirmed by the fact that the $1/\chi_p$ curve goes exactly to the origin and the $\mu_{eff}(T)$ dependence does not exhibit any singularity upon approaching $T = 0$ (Figure S8b). The effective moment calculated for the case of non-interacting ions ($\theta = 0$) reaches $\mu_{eff} \approx 2.87\ \mu_B$ at room temperature (Figure S8b), which agrees well with the values observed usually for samarium Sm^{3+} ions, $\mu_{eff}(Sm) \approx 1.5\ \mu_B$, and copper Cu^{2+}, $\mu_{eff}(Cu) \approx 2\ \mu_B$: $\mu_{eff} \approx \sqrt{2\cdot 1.5^2 + 2^2} \approx 2.92\ \mu_B$. As the temperature decreases, μ_{eff} decreases smoothly from ~2.87 μ_B down to ~2.0 μ_B at T = 1.77 K. Most likely, the effective-moment reduction originates from the transition of Sm^{3+} ions to the doublet ±1/2 ground state which is weakly magnetic due to the small g factor. Consequently, the magnetic susceptibility of the Sm-Cu complex at low temperature is determined mainly by the contribution of Cu^{2+} ions with a small addition from Sm^{3+} ones, while at higher temperatures excited doublets of the Sm^{3+} ground-state multiplet J = 5/2 as well as levels of other multiplets come into play resulting in the increase of the Sm^{3+} contribution [*1].

***La-Co complex* (5)**

The La-Co complex exhibits paramagnetic behavior throughout the available temperature range without any magnetic phase transition or manifestation of inter-ion interactions (Figure S9a). The absence of significant inter-ion interactions is confirmed by the $1/\chi_p$ curve tending to zero at $T \approx 0$ and the $\mu_{eff}(T)$ dependence approaching zero temperature without singularity (Figure S9b). The effective magnetic moment measured in the field H = 1 kOe varies from $\mu_{eff} \approx 4.73\ \mu_B$ at room temperature to $\mu_{eff} \approx 3.8\ \mu_B$ at T = 1.77 K. Both high- and low-temperature μ_{eff} values are typical of Co^{2+} (S = 3/2) complexes. At high temperature, μ_{eff} significantly exceeds the theoretical spin-only value of 3.87 μ_B due to the contribution of orbital moments. At low temperature, the effective magnetic momentum decreases both due to the reduction of the orbital-moment contribution and as a result of zero-field splitting of the Co^{2+} multiplet by the crystal field [*2]. It should be noted that the sharp drop in μ_{eff} measured in the field H = 10 kOe seen at low temperatures is not a manifestation of exchange interactions but is caused by the nonlinearity of the magnetization $M(H)$ curve, typical for Co^{2+} ions (S = 3/2) (Figure S9c). Thus, from the point of view of magnetic properties the La-Co complex can be considered as a set of almost non-interacting Co^{2+} ions (S = 3/2).

a

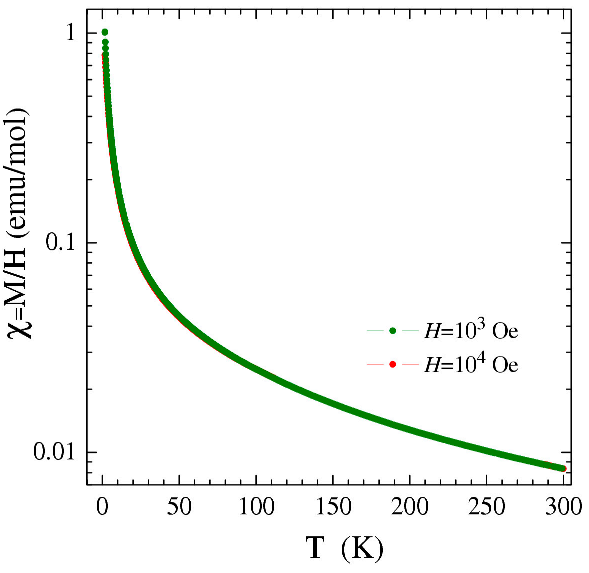

b

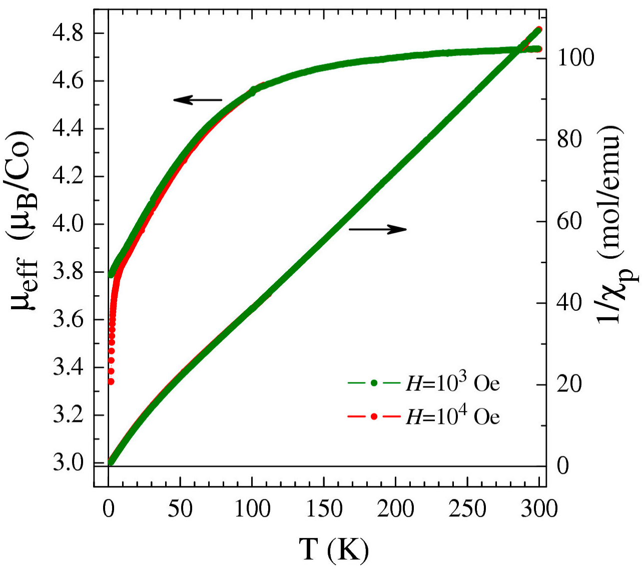

c

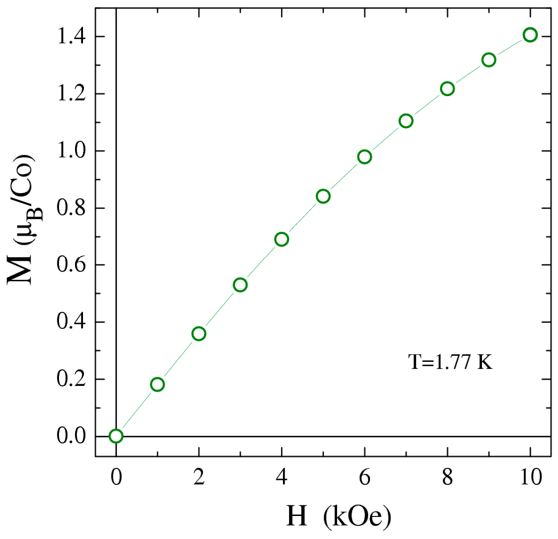

Figure S9: (a) Temperature dependence of the magnetic susceptibility χ measured for the La-Co complex at the magnetic fields H = 1; 10 kOe. (b) Temperature dependences of μ_{eff} and $1/\chi_p$. The depicted effective moment μ_{eff} is calculated for the case of non-interacting magnetic moments (θ = 0). (c) Magnetic field dependence of the magnetization measured at T = 1.77 K.

La-Ni complex (6)

The La-Ni complex exhibits paramagnetic behavior throughout the available temperature range (Figure S10), but at the lowest temperatures the paramagnetic susceptibility growth slows noticeably, indicating a transition to a weakly magnetic state. The calculated effective magnetic moment decreases smoothly from $\mu_{\mathrm{eff}} \approx 3.15\ \mu_{\mathrm{B}}$ at room temperature down to $\mu_{\mathrm{eff}} \approx 1.1\ \mu_{\mathrm{B}}$ at T = 50 K. The high-temperature value of μ_{eff} is close to the theoretical spin-only value of 2.83 μ_{B} for Ni^{2+} (S = 1); the slight excess is due to the contribution of orbital moments. The smooth decrease of the effective moment upon cooling is associated with the transition of Ni^{2+} ions into the weakly magnetic (singlet) ground state as a result of zero-field splitting of the S = 1 multiplet [*2] or formation of the low-spin S = 0 state.

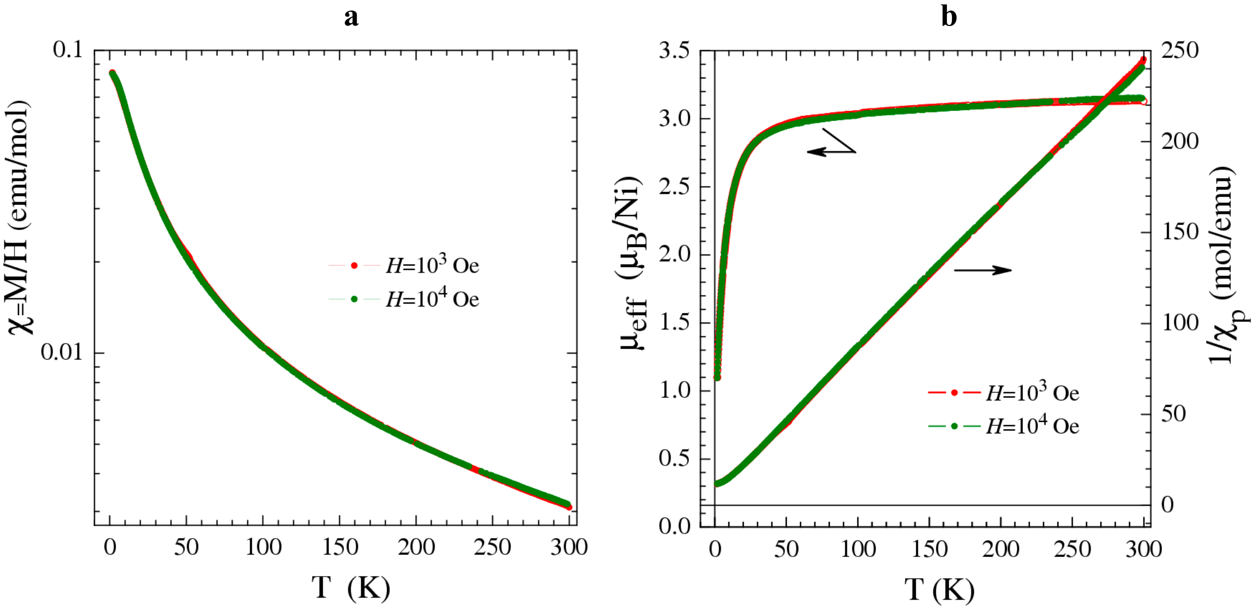

Figure S10: (a) Temperature dependence of the magnetic susceptibility χ measured for the La-Ni complex at the magnetic fields H = 1; 10 kOe. (b) Temperature dependences of μ_{eff} and $1/\chi_{\mathrm{P}}$. The depicted effective moment μ_{eff} is calculated for the case of non-interacting magnetic moments (θ = 0).

[*1] A. K. Zvezdin, V. M. Matveev, A. A. Mukhin, and A. I. Popov, *Rare Earth Ions in Magnetically Ordered Crystals* (Nauka, Moscow, 1985).
[*2] R. Boča, Coord. Chem. Rev. **248**, 757 (2004).
